# Synthesis, Characterization, and Flocculation Studies of β‐Cyclodextrin‐Based Stimuli‐Responsive Star Copolymer: An Environmental Remediation

**DOI:** 10.1002/gch2.201900089

**Published:** 2020-04-01

**Authors:** Kiran, Rambabu Koyilapu, Rudramani Tiwari, Subramanian Krishnamoorthi, Krishna Kumar

**Affiliations:** ^1^ Department of Chemistry and Environmental Science Madan Mohan Malaviya University of Technology Gorakhpur 273010 India; ^2^ School of Chemistry University of Hyderabad Gachibowli Hyderabad 500046 India; ^3^ Department of Chemistry Institute of Science Banaras Hindu University Varanasi 221005 India

**Keywords:** block copolymers, β‐cyclodextrin, flocculation, RAFT polymerization, thermoresponsive polymers

## Abstract

Thermoresponsive star polymers are synthesized by using poly(NIPAM‐*b*‐DMA) diblock copolymer and β‐cyclodextrin (β‐CD). The synthesis of thermoresponsive diblock copolymer (TRP) is carried out by reversible addition–fragmentation chain transfer mediated aqueous polymerization using *N*‐isopropylacrylamide (PNIPAM) and di‐methylacrylamide as monomers. Mercaptopropionic acid is used as in situ chain transfer agent (CTA) to synthesize PNIPAM, i.e., macro‐CTA. The polymeric materials are characterized by Fourier‐transform infrared spectroscopy, gel permeation chromatography, ^1^H‐NMR, particle size measurement, scanning electron microscopy, thermogravimetric analysis, UV–vis spectroscopy, and X‐ray diffraction analysis. PNIPAM, TRP, and *b*‐CD grafted thermoresponsive diblock copolymer (β‐CD‐TRP) show lower critical solution temperature at 32.8, 34.3, and 36.8 °C, respectively. TRP and β‐CD‐TRP are also studied for the removal of model contaminant (kaolin). Among all grades, β‐CD‐TRP 4 shows the best performance in the removal of kaolin from aqueous solution at 25 and 50 °C.

## Graphical abstract

The enhanced flocculation performance of thermoresponsive diblock copolymer (TRP) and *b*‐CD grafted thermoresponsive diblock copolymer *b*‐CD‐TRP is demonstrated below and above lower critical solution temperature (LCST) in kaolin suspension at neutral pH.

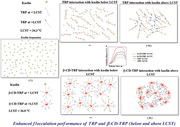

## 1 Introduction

For the survival of human beings and any modern industry fresh water is an essential factor. To meet the requirements of fresh/potable water, the immediate call is to treat wastewater, especially the municipal and industrial effluents. These effluents are highly undesirable and unsafe. In the past decades, several studies have been conducted in order to promote the research work in the field of wastewater treatment which involves the process of coagulation and flocculation.

Organic polymeric flocculants (OPFs) attract more attention toward wastewater treatment due to its fast, effective, and efficient performance. OPFs have higher molecular weight (*M*_w_), linear versus branched architecture, ionic nature, diverse chemical composition, and various functional groups as substituents in polymer backbone,^[^ ^1^ ^]^ which are highly efficient with little doses (e.g., few ppm) and generate much lesser sludge volume without consumption of alkalinity unlike inorganic coagulants. The flocs formed during flocculation are moreover bigger and stronger to endow excellent settling behavior.^[^ ^1^, ^2^ ^]^

Recently, temperature‐responsive star, homopolymer, and diblock copolymer based on poly(*N*‐isopropylacrylamide) (P(NIPAM)) have been reported as novel flocculant to accelerate settling rate and enhanced flocculation.^[^ ^3^, ^4^, ^5^, ^6^, ^7^, ^8^, ^9^, ^10^, ^11^, ^12^ ^]^

Temperature‐responsive polymers are soluble in aqueous solutions below their lower critical solution temperature (LCST) and insoluble above LCST due to phase transition behavior.^[^ ^13^, ^14^, ^15^, ^16^ ^]^ As a result, suspended particles/contaminants in tailings suspension are rapidly flocculated and settled due to those strong hydrophobic interactions. Li and co‐workers analyzed the interactive (adhesion) force between P(NIPAM) and kaolin particles by atomic force microscopy. The reports showed that the interactive force augmented from 0 to 3.5 mN m^−1^ by raising temperature from ambient to 40 °C, and the repulsion between kaolin particles reduced to almost zero.^[^ ^13^ ^]^

RAFT polymerization of NIPAM‐ and NIPAM‐based diblock copolymers in water through chain transfer agents (CTAs) is more difficult than expected because the resultant PNIPAM‐based diblock copolymers have a stimuli‐sensitive LCST. As discussed in many reports, the LCST of the PNIPAM‐based diblock copolymers is directly proportional to the chain length of the hydrophilic block and the PNIPAM block.^[^ ^17^, ^18^, ^19^, ^20^, ^21^, ^22^, ^23^ ^]^

Poly(*N*,*N*‐dimethylacrylamide) (PDMA) is hydrophilic polymer and its many applications are reported in wastewater treatment and in pharmaceuticals.^[^ ^24^ ^]^ Chemistry behind the PDMA is that it has two methyl groups at the “N” atom, which increases the electron density and hence polarity of the amide functionality. In the case of PDMA polymeric chain, the methyl disubstituted –NH_2_ groups do not take part in intramolecular hydrogen bonding with the carbonyl groups of the neighboring amide groups that slightly enhance the water solubility.^[^ ^25^ ^]^

In this work, block copolymers have been synthesized by using NIPAM and DMA monomers. The LCST of polymer has been increased by 4 °C by using DMA. Then, the postpolymerization changes have been carried out to make the polymer biodegradable and eco‐friendly by using β‐cyclodextrin (β‐CD are essentially composed of α‐1,4‐linked d(α)‐glucopyranose oligosaccharides). Addition of β‐CD further tailors the polymer in star shape which results in increased molecular weight and decreased Nephelometric Turbidity Unit (NTU) of wastewater. NTU has been determined by a jar test method in which kaolin suspension solution has been taken as a probe. So, this diblock copolymer and star polymer can be proved as successful and biodegradable materials for wastewater treatment in the days to come.

## 2 Materials and Method

Dimethylacrylamide (DMA) was procured from Alfa Aesar, India. β‐Cyclodextrin (β‐CD), *N*‐isopropylacrylamide (NIPAM), and azobis‐isobutyronitrile (AIBN) were purchased from Sigma‐Aldrich. 3‐Mercaptopropionic acid (MCPA) was received from Alfa Aesar. NIPAM was recrystallized in hexane, and DMA and AIBN were recrystallized in methanol prior to use. 1‐Ethyl‐3‐(3‐dimethylaminopropyl) carbodiimide (EDC) and *N*‐hydroxysuccinimide (NHS) were purchased from TCI, India. Kaolin was received from Himedia, India. Diethyl ether, dimethyl sulfoxide (DMSO), and tetrahydrofuran (THF) were received from Fisher Scientific, India.

### 2.1 Experimental Section

#### 2.1.1 Synthesis of Poly(*N*‐Isopropylacrylamide)

**Scheme 1:**
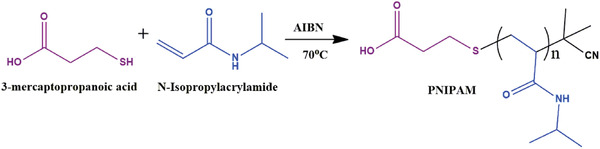
Synthetic route of macro‐CTA.

The synthesis of PNIPAM (macro‐CTA) was carried out by using *N*‐isopropylacrylamide and MCPA (mercaptopropionic acid) followed by the RAFT polymerization technique. For the synthesis of PNIPAM, 100 mg of mercaptopropionic acid was taken in Schlenk tube and dissolved in 10 mL of THF and then added 3 g of *N*‐isopropylacrylamide under the nitrogen atmosphere. Afterward, the Schlenk tube was placed in the preheated oil bath of 60 °C and further charged by AIBN (0.05 mmol). When the solution became viscous and stirring was slow down, the polymerization was quenched by the mixture of acetone and diethyl ether (1:1) and polymer was precipitated. Then, the polymer was kept in a vacuum oven (15 mm of Hg) for 24 h at ambient temperature. After this, the dried polymer had been pulverized and used for characterization/applications. GPC data of PNIPAM are weight average molecular weight (*M*_w_) = 32 312, number average molecular weight (*M*_n_) = 29 126, and polydispersity index (PDI) = 1.10938. The GPC curve is shown in Figure S1 (Supporting Information). The synthetic route of β‐CD‐based macro‐CTA (I) is shown in **Scheme** **1**.

#### 2.1.2 Synthesis of Thermoresponsive Diblock Copolymer (TRP)

**Scheme 2:**
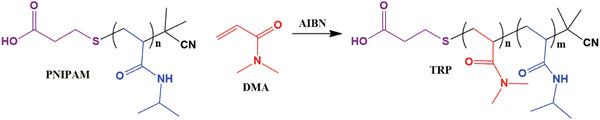
Synthetic route of diblock copolymers.

Thermoresponsive diblock copolymers (TRP) have been synthesized by using PNIPAM as macro‐CTA, DMA as monomer, and AIBN as initiator through the RAFT polymerization technique. Five different grades of TRP from series 1 to 5 were synthesized by varying the amount of DMA and remaining reaction feed was kept same in THF. Synthetic details of TRP are summarized in **Table** **1**. The synthesis and processing of polymer grades was carried out by the above‐mentioned procedure. The synthetic route of diblock copolymer (II) is shown in **Scheme** **2**.

**Table 1:** Synthetic details of TRP

S. No.	Series	PNIPAM [g]	DMA [g]	AIBN [mmol]	Yield [%]	GPC data		
						*M*_w_	*M*_n_	PDI
1.	TRP 1	1	1	0.05	89	64 156	44 009	1.4577
2.	TRP 2	1	3	0.05	78	85 907	74 936	1.1464
3.	TRP 3	1	5	0.05	91	108 699	104 980	1.0354
4.	TRP 4	1	7	0.05	88	158 644	149 911	1.0582
5.	TRP 5	1	9	0.05	83	115 525	111 812	1.0332

**Table 2:** Synthetic details of β‐CD‐TRP

S. No.	Grades	TRP [g]	β‐CD [mg]	EDC	NHS	Yield [%]	GPC data		
							*M*_w_	*M*_n_	PDI
1.	β‐CD‐TRP 1	01	10	100	100	93	305 422	303 791	1.0053
2.	β‐CD‐TRP 2	01	10	100	100	91	539 625	470 085	1.1479
3.	β‐CD‐TRP 3	01	10	100	100	95	600 406	597 915	1.0041
4.	β‐CD‐TRP 4	01	10	100	100	89	905 748	747 390	1.2118
5.	β‐CD‐TRP 5	01	10	100	100	78	799 979	668 106	1.1973

**Scheme 3:**
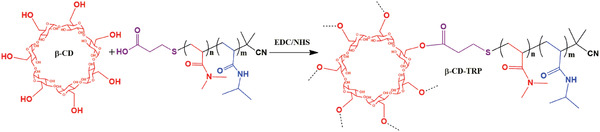
Synthetic route of β‐CD‐TRP.

#### 2.1.3 Synthesis of β‐CD‐TRP

β‐CD‐TRP was synthesized by adding 10 mg of β‐CD in all the five grades of TRP. And, this has been performed by taking 01 g of TRP in a round bottom flask dissolved in a solution of DMSO:water (1:1). This RB flask was kept under stirring and continuous nitrogen purging. To this flask, 100 mg of EDC and NHS each were added. This solution was kept under stirring for 24 h, after that the solution mixture was poured in a suitable cutout dialysis membrane and stirred for 3 days in double distilled water. Afterward, the reaction mixture was precipitated by using the equal amount of acetone and diethyl ether in the ratio of 1:1. Then, the precipitates were filtered and dried under vacuum. Synthetic details of β‐CD‐TRP are summarized in **Table** **2**. A similar method was used for four other grades. The synthetic route of β‐CD‐TRP is shown in **Scheme** **3**.

### 2.2 Instrumentation

#### 2.2.1 FT‐IR

Fourier transform infrared spectra of PNIPAM, TRP, and β‐CD‐TRP were recorded by spectrophotometer Perkin Elmer (spectrum 2) within the range of 4000–400 cm^−1^ by using a solid‐state KBr pellet method.

#### 2.2.2 ^1^H‐NMR

Proton nuclear magnetic resonance spectrometer FT‐NMR JEOL AL 500 FT‐NMR was used to record ^1^H‐NMR of polymers in DMSO. Tetramethylsilane was used as an internal reference.

#### 2.2.3 GPC

Molecular weight parameters (*M*_n_, *M*_w_, and PDI) of polymers were determined by Waters 2414 (refractive index detector) gel permeation chromatography (GPC) in water at 40 °C with a flow rate of 0.5 mL min^−1^ on 1000 ultrahydrogel columns connected to a Waters 515 HPLC Pump using Breeze Software. The columns were calibrated against five polyethylene oxide narrow standard samples. The sample filter pore size of 0.45 µm was used to filter the polymer samples.

#### 2.2.4 LCST Measurement

UV–vis spectrophotometer (Shimadzu UV‐1700 Pharmaspec from Kyoto, Japan) was engaged in the estimation of the LCST value of PNIPAM, TRP, and β‐CD‐TRP. The UV scan was observed at different temperatures for all polymer samples at 280 nm.

#### 2.2.5 Particle Size Measurement

Particle size measurements of PNIPAM, TRP, and β‐CD‐TRP (at below and above LCST) were performed using a Zetasizer Nano S90 (Malvern Instruments, Germany) operating at 4 mW He–Ne laser with 633 nm wavelength.

#### 2.2.6 SEM

Scanning electron microscope HRSEM SUPRA 40, ZEISS (Germany) was used for SEM images of PNIPAM, TRP, and β‐CD‐TRP in the powder form.

#### 2.2.7 TG Analysis

The thermogravimetric analysis of PNIPAM, TRP, and β‐CD‐TRP was carried out by TGA (METTER‐TOLEDO) from ambient to 500 °C, under nitrogen atmosphere. The heating rate was 10 °C min^−1^.

#### 2.2.8 XRD

X‐ray diffraction analysis of PNIPAM, TRP, and β‐CD‐TRP was performed by 18KW Cu‐rotating anode RIGAKU (Tokyo, Japan). The scattering angle (2θ) was varied from 5° to 70°.

### 2.3 Application

#### 2.3.1 Turbidity Removal Studies

##### Flocculation Performance in Kaolin Suspension Using a Jar Test Method

The jar test method was used to analyze the flocculation performance of TRP and β‐CD‐TRP in 0.25% w/v kaolin suspension in water with 25 and 50 °C at neutral pH. Six‐jar batch flocculator supplied by M.B. Flocculators, Mumbai, India, was used for the jar test. The jar test method was followed as in our previous published article.^[^ ^26^ ^]^ The turbidity of treated water was determined in the Nephelo Turbidity Unit (NTU) by using a digital Nephelometer.

## 3 Result and Discussion

### 3.1 Synthesis of PNIPAM, TRP, and β‐CD‐TRP

The synthesis of PNIPAM has been successfully carried out by the one‐pot synthesis method with good yield and it has proved to be an effective and efficient macro‐CTA. Many attempts for the synthesis of PNIPAM were targeted by using different oligosaccharide‐ and polysaccharide‐based CTA, but get failed (low molecular weight polymer on the order of 10^3^ and lesser yield). Then, MCPA was used for synthesis and the desired polymer was obtained. The advantage of MCPA over the traditional CTA is that MCPA forms in situ CTA during polymerization and results are also effective and efficient. MCPA‐based macro‐CTA consists active carboxylic functional groups at the terminal of the polymer.

Thermoresponsive diblock copolymers (TRP) were successfully synthesized by using PNIPAM (macro‐CTA) and the grades (controlled and higher in molecular weight) were optimized by varying the amount of DMA. The GPC curve of all TRP is shown in Figures S2–S6 (Supporting Information). TRP grade 4 shows the higher yield and molecular weight among all grades. From the initial to final grades, the polymerization was performed well but the molecular weight increased up to grade 4 and after that it decreased. This can be attributed to the fact that the presence of higher monomer concentration results in increase in the viscosity of the solution which results in lowering of monomer conversion. Polymerization kinetics studies have not been performed because of the initial higher viscosity of reaction mixtures.

Further, five different grades of star copolymers (β‐CD‐TRP) were formulated by using the above‐synthesized grades (TRP). And, the condensation reaction was performed between the β‐cyclodextrin hydroxyl group and terminal carboxylic group of the diblock copolymer.

β‐CD‐TRP 4 shows the higher molecular weight. The GPC curve of all thermoresponsive star polymers is shown in Figures S7–S11 (Supporting Information).

### 3.2 FT‐IR

**Figure 1:**
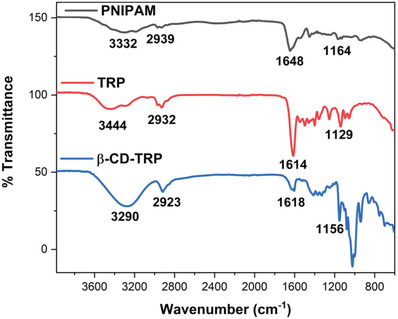
FT‐IR spectra of PNIPAM, TRP, and β‐CD‐TRP.

FT‐IR spectra of PNIPAM, TRP, and β‐CD‐TRP are depicted in **Figure** **1**. In the FT‐IR spectrum of PNIPAM, the absorption band of asymmetric –NH was observed at 3332 cm^−1^, the –CH absorption band was depicted at 2939 cm^−1^, the combination of amides I and II was observed at 1648 cm^−1^, and the –C–O absorption peak was observed at 1164 cm^−1^. These characteristic peaks have confirmed that the PNIPAM synthesis has been successfully carried out.

The characteristic absorption bands of PNIPAM and DMA were observed in the TRP spectrum. The asymmetric –NH of PNIPAM was attributed at 3444 cm^−1^, the combination of the –CH broad absorption band of PNIPAM and DMA was observed at 2932 cm^−1^, the combination of amides I and II of both parts was observed at 1614 cm^−1^, and the –C–O absorption peak was attributed at 1129 cm^−1^.

In the FT‐IR spectrum of β‐CD‐TRP, the combination of asymmetric –NH (PNIPAM) and –OH (β‐CD) absorption band was observed at 3290 cm^−1^, the combination of –CH broad absorption band of PNIPAM, DMA, and β‐CD was depicted at 2923 cm^−1^, the combination of amides I and II was observed at 1618 cm^−1^, and the –C–O absorption peak was observed at 1156 cm^−1^. The observed characteristic peak confirms that the synthesis of β‐CD‐TRP has been successfully carried out.

### 3.3 ^1^H‐NMR

**Figure 2:**
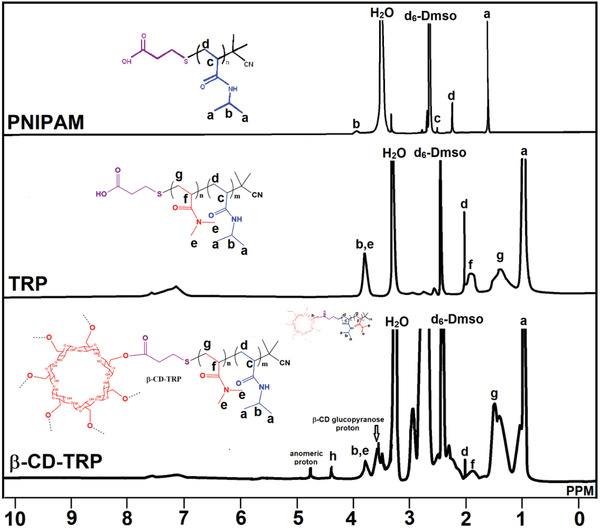
^1^H‐NMR spectra of PNIPAM, TRP, and β‐CD‐TRP.

^1^H‐NMR spectra of PNIPAM, TRP, and β‐CD‐TRP are shown in **Figure** **2**. In the ^1^H‐NMR spectrum of PNIPAM (DMSO, 500 MHz), methyl proton signal was depicted at δ 1.38 ppm (H_a_, 6H), tertiary C proton signal was observed at δ 3.84 ppm (H_b_, 1H), proton signals of methylene and methyne groups were observed at δ 2.10 ppm (H_d_, 2H) and δ 3.16 ppm (H_c_, 1H), respectively. In the ^1^H‐NMR spectrum of TRP (DMSO, 500 MHz), methyl proton signal of PNIPAM was depicted at δ 1.04 ppm (H_a_, 6H), methylene proton signals of PNIPAM and DMA were observed at δ 2.02 ppm (H_d_, 2H) and δ 1.47 ppm (H_g_, 2H) respectively, methyne proton peak of DMA was observed at δ 1.88 ppm (H_f_, 1H), and methyne proton peak of PNIPAM was overlapped with solvent peak. Combination of the methyl group of DMA and tertiary carbon proton signal was depicted at δ 3.80 ppm (H_b&e_). These characteristic signals confirm that TRP was synthesized successfully.

Methylene groups adjacent to xanthate groups were observed at δ 4.38 ppm (H_5_), other β‐CD signals were observed at δ 3.29–3.67 ppm (H_4&7–9_), and methyne and methylene proton signals of PAM were attributed at δ 1.84 ppm (H_11_) and δ 1.09 ppm (H_10_), respectively. These signals confirm that the grafting of PAM has been successfully carried out on the β‐CD backbone.

In the ^1^H‐NMR spectrum of β‐CD‐TRP (DMSO, 500 MHz), methyl proton signal of PNIPAM was depicted at δ 1.07 ppm (H_a_, 6H), methylene proton signals of PNIPAM and DMA were observed at δ 2.04 ppm (H_d_, 2H) and δ 1.27 ppm (H_g_, 2H) respectively, methyne proton peak of DMA was observed at δ 1.89 ppm (H_f_, 1H), and methyne proton peak of PNIPAM was overlapped with solvent peak. Combination of the methyl group of DMA and tertiary carbon proton signal was depicted at δ 3.80 ppm (H_b&e_). Multiplet proton signal (δ 3.36–3.65 ppm) was attributed to β‐CD glucopyranose proton. These characteristic proton signals confirm that the grafting of TRP has been successfully carried out on the β‐CD backbone.

### 3.4 LCST Measurement

**Figure 3:**
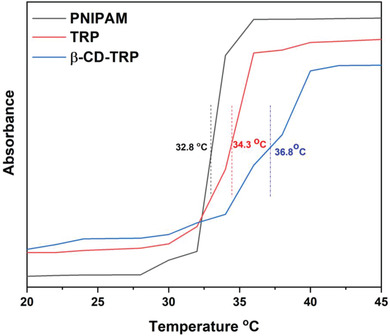
LCST measurement of PNIPAM, TRP, and β‐CD‐TRP.

The LCST value of PNIPAM, TRP, and β‐CD‐TRP was determined by plotting the graph of temperature versus absorbance. From 20 to 32 °C, the absorbance is less which signifies that all polymers are soluble in aqueous medium. Afterward, the absorbance increases gradually at different temperatures and represents that the polymer becomes hydrophobic. In the above given graph, LCST of PNIPAM, TRP, and β‐CD‐TRP is depicted at 33.0, 34.3, and 36.8 °C. **Figure** **3** shows the LCST measurement of PNIPAM, TRP, and β‐CD‐TRP.

### 3.5 Particle Size Measurement

PNIPAM, TRP, and β‐CD‐TRP particle sizes were measured by light scattering, signifying the monodispersity of the formed particles. At 25 °C, the size of PNIPAM, TRP, and β‐CD‐TRP is 255, 342, and 459 nm and at 50 °C the size is 190, 531, and 955 nm, respectively.

**Figure 4:**
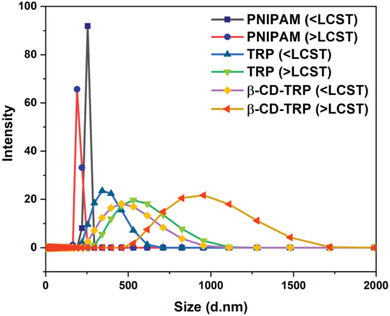
Particle size measurements using light scattering of PNIPAM, TRP, and β‐CD‐TRP (below and above LCST).

In the case of PNIPAM, the particle size decreases above LCST because of the hydrophobic behavior of polymer due to its thermoresponsive properties. TRP and β‐CD‐TRP size increases above LCST due to amphiphilic nature (i.e., above LCST PDMA part remains hydrophilic while PNIPAM part becomes hydrophobic) and it gets coagulated. Particle size measurement is shown in **Figure** **4**.

### 3.6 SEM

**Figure 5:**
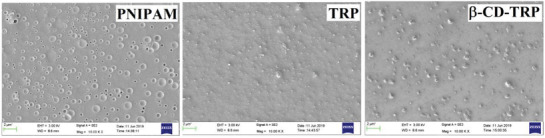
SEM image of PNIPAM, TRP, and β‐CD‐TRP.

SEM images of PNIPAM, TRP, and β‐CD‐TRP are shown in **Figure** **5**. The SEM image of PNIPAM shows the porous nature of surface with pore size ranging from 124.7 to 199.1 nm. The SEM image of TRP shows that the surface is filled with some coarse material and porous nature has been disappeared. In the case of β‐CD‐TRP, the coarseness of surface is enhanced due to the addition of β‐CD and the surface becomes rough. Thus, SEM analysis supports the morphology changes with respect to the modifications carried out.

### 3.7 TG Analysis

**Figure 6:**
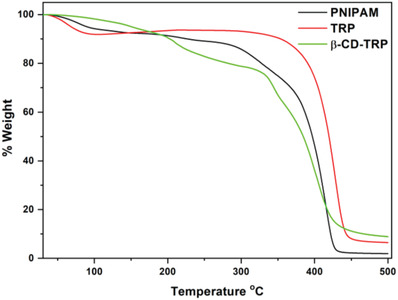
TGA pattern of PNIPAM, TRP, and β‐CD‐TRP.

It shows the thermal degradation pattern of PNIPAM, TRP, and β‐CD‐TRP. PNIPAM and TRP show the first weight loss at 150 °C due to the loss of absorbed moisture. The second weight loss was due to the breaking of functional groups and was observed at 300 and 350 °C, respectively, for PNIPAM and TRP, and the third degradation was observed at 430 and 450 °C for both due to the breakage of polymer backbone. However, in the case of β‐CD‐TRP, the first weight loss was observed at 200 °C due to the loss of absorbed moisture. The second weight loss was observed at 330 °C due to the breaking of bonds between β‐CD and polymer functionality. The third hump is showing degradation at 440 °C due to the breakage of polymer structure. PNIPAM, TRP, and β‐CD‐TRP are thermally stable up to 150 °C. The TGA pattern of PNIPAM, TRP, and β‐CD‐TRP is shown in **Figure** **6**.

### 3.8 XRD

**Figure 7:**
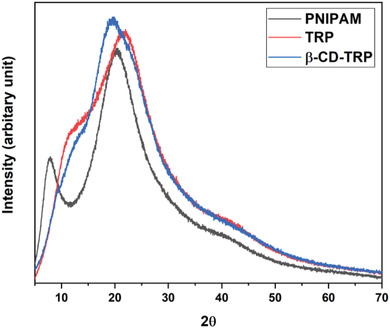
XRD analysis of PNIPAM TRP and β‐CD‐TRP.

The XRD analysis of PNIPAM, TRP, and β‐CD‐TRP is shown in **Figure** **7**. The XRD analysis shows that all polymers have amorphous nature. The powder XRD patterns of PNIPAM display distinct peaks at 2θ values of about 20.2 and 7.6. TRP and β‐CD‐TRP show the 2θ peaks at 21.8 and 11.5 and 19.6 and 12.7, respectively. The observed peaks of PNIPAM were carried forward in the XRD pattern of TRP and β‐CD‐TRP, and it confirms that PNIPAM is well connected in TRP and β‐CD‐TRP frame.

### 3.9 Turbidity Removal Studies

#### 3.9.1 Jar Test

**Figure 8:**
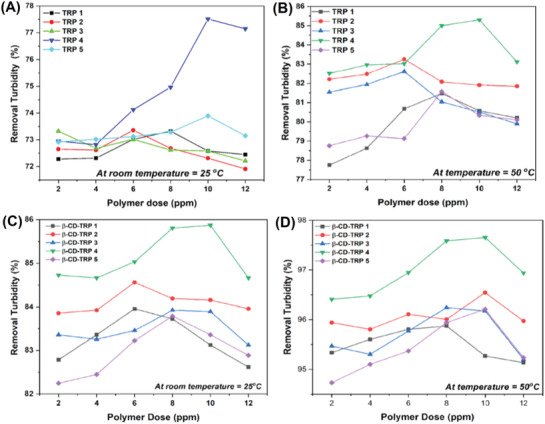
The jar test results showing turbidity removal percentage.

Flocculation performances of polymers were analyzed by the jar test method. The jar test performance of TRP (grades 1–5) and β‐CD‐TRP (grades 1–5) in kaolin suspension solution of water at 25 and 50 °C is depicted in **Figure** **8**A–D, respectively. Figure 8 shows the turbidity removal (%) plotted against polymer dose (2–12 ppm) of all grades.

At room temperature, in the case of TRP flocculation occurs by interaction of colloidal particles with polymer chains. The arms of TRP are floating in water and interact with impurities. These arms capture the impurities and settle down due to increasing weight polymer–colloidal particle matrix. But the least interacted polymer–colloidal particle matrix stays dispersed in water due to the hydrophilic nature of polymer and therefore desired purification (100%) cannot be achieved. The percentage of turbidity removal in this case is 77.7% at 10 ppm of polymer dosage.

However, in the case of β‐CD‐TRP, β‐CD acts as a hydrophobic inner core due to which the dispersion volume of polymer in solution becomes more as compared to TRP. So, the polymer would interact more with impurities and the entrapment will be high resulting in the formation of bigger flocs. Ultimately, these flocs settle down and overall turbidity removal percentage will be increased, i.e., 85.9% at 10 ppm of polymer dosage.

At 50 °C further flocculation was carried out of all the grades of TRP and β‐CD‐TRP. It is well known that TRP consists of two parts (i.e., PDMA and PNIPAM) and β‐CD‐TRP consists of three parts (i.e., β‐CD, PDMA, and PNIPAM). On heating, the hydrophobic behavior of β‐CD and hydrophilic behavior of PDMA remain intact but the nature of PNIPAM changes and its arm would get coagulated and therefore the polymer would not remain effectively dispersed in water as it was earlier. Now the interactions of polymer with the solution become lesser and instead of floating the polymer settle down faster at the bottom. In a similar manner on interacting with polymer all the molecules of impurities come closer to each other forming bigger flocs and settle down at bottom of the jar.

**Figure 9:**
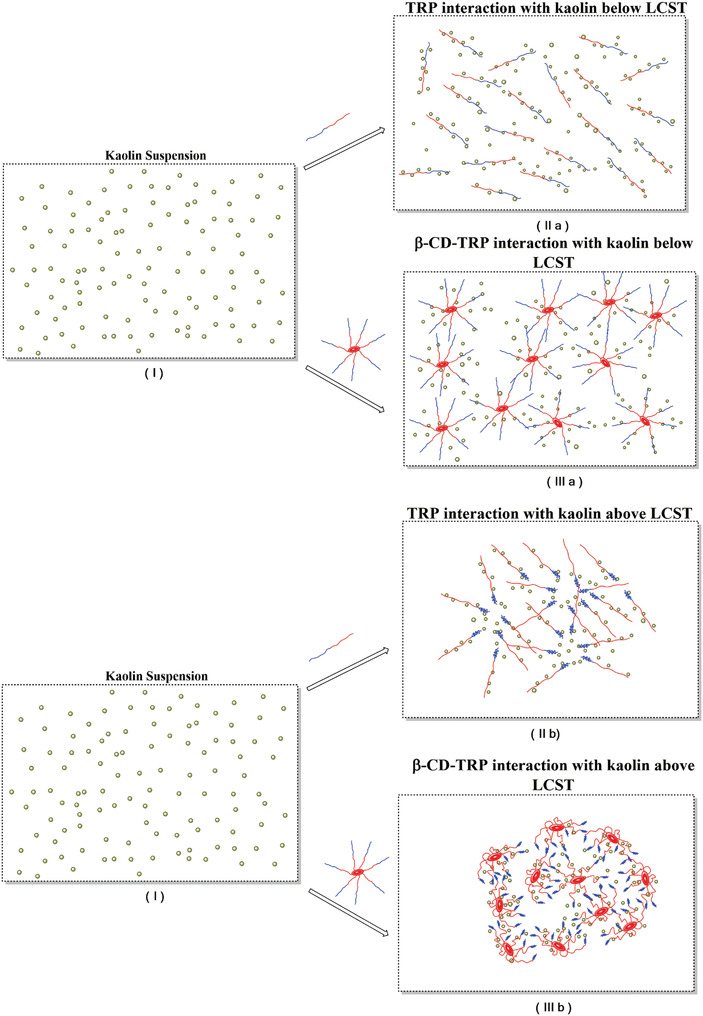
a) Panel I shows the kaolin suspension and panels IIa and IIIa show the physiochemical interaction between polymer and kaolin of TRP and β‐CD‐TRP below LCST. b) Panel I shows the kaolin suspension and panels IIb and IIIb show the physiochemical interaction between polymer and kaolin in TRP and β‐CD‐TRP above LCST.

Among the grades of TRP and β‐CD‐TRP, grade 4 shows the highest turbidity removal, i.e., 85.3% and 97.5%, respectively, at 10 ppm of polymer dosage above LCST as shown in **Figure** **9**b.

## 4 Conclusion

Five different grades of TRP were synthesized through block copolymerization of NIPAM and DMA using the RAFT polymerization technique. Then, the β‐CD was connected to TRP to study the postpolymerization changes and provided biodegradable backbone. These changes were proved and characterize by FT‐IR and ^1^H‐NMR analysis. The physical properties of TRP and β‐CD‐TRP were observed from SEM and XRD for crystallinity alteration. β‐CD‐TRP were found to be thermally more stable than that of TRP through TGA analysis. Thermoresponsive behavior of PNIPAM, TRP, and β‐CD‐TRP was successfully confirmed by particle size measurement and UV–vis spectroscopy. Among the five grades, grade 4 of TRP and β‐CD‐TRP showed the best solid liquid separation (highest turbidity removal at lower doses) at room and high temperatures in a jar test. Thus, β‐CD‐TRP are proved to be the intelligent materials for colloidal particle removal from wastewater. Also, these findings can meet the challenge of water purification in industries.

## Conflict of Interest

The authors declare no conflict of interest.

## Supporting information

Supporting Information
