# Supplementary material for: Synthesis, Characterization, and Flocculation Studies of β‐Cyclodextrin‐Based Stimuli‐Responsive Star Copolymer: An Environmental Remediation
Source: Glob Chall. 2020 Apr 1;4(7):1900089. doi: 10.1002/gch2.201900089 (PMC7330502; doi:10.1002/gch2.201900089)

Copyright WILEY-VCH Verlag GmbH & Co. KGaA, 69469 Weinheim, Germany, 2020.

Global Challenges
Open Access

Supporting Information

for *Global Challenges,* DOI: 10.1002/gch2.201900089

Synthesis, Characterization, and Flocculation Studies of #-Cyclodextrin-Based Stimuli-Responsive Star Copolymer: An Environmental Remediation

*Kiran, Rambabu Koyilapu, Rudramani Tiwari, Subramanian Krishnamoorthi, and Krishna Kumar**

Synthesis, characterization and flocculation studies of β-Cyclodextrin based stimuli- responsive star copolymer: An Environmental remediation

Kiran[a], Rambabu Koyilapu,[c] Rudramani Tiwari,[b] S. Krishnamoorthi[b] and Krishna Kumar[a]*

[a]*Applied Science Department, Madan Mohan Malaviya University of Technology, Gorakhpur-273010, India*
[b]*Department of Chemistry, Institute of Science, Banaras Hindu University, Varanasi-221005, India*
[c]*School of Chemistry, University of Hyderabad, Gachibowli, Hyderabad-500046, India*

***Corresponding author's email id:** neerajrab@gmail.com*

GPC DATA

Supporting Figure (SF)

SF 1: PNIPAM

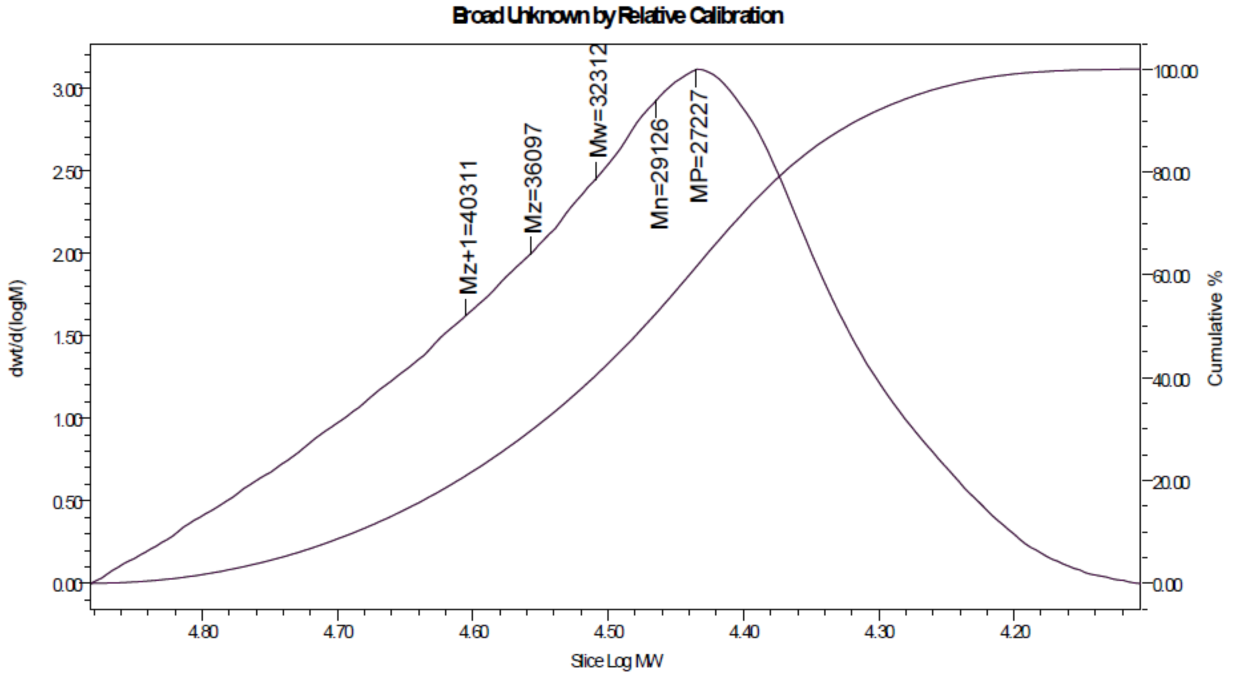

SF 2: TRP-1

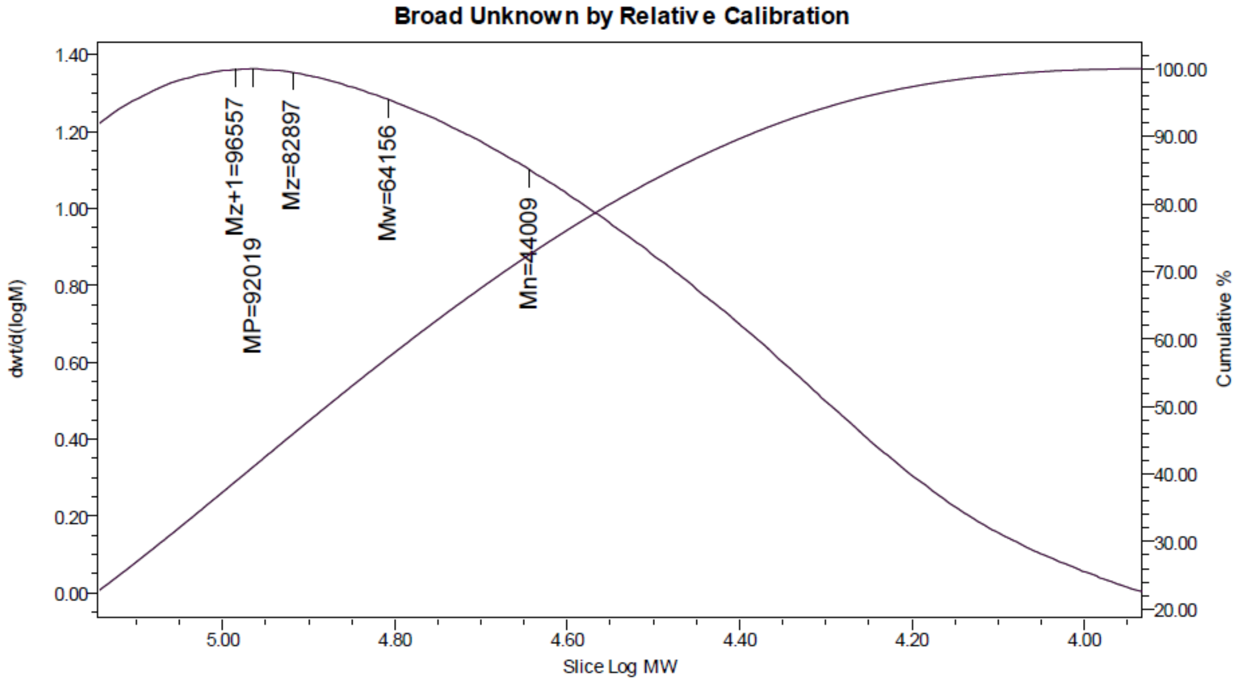

SF 3: TRP-2

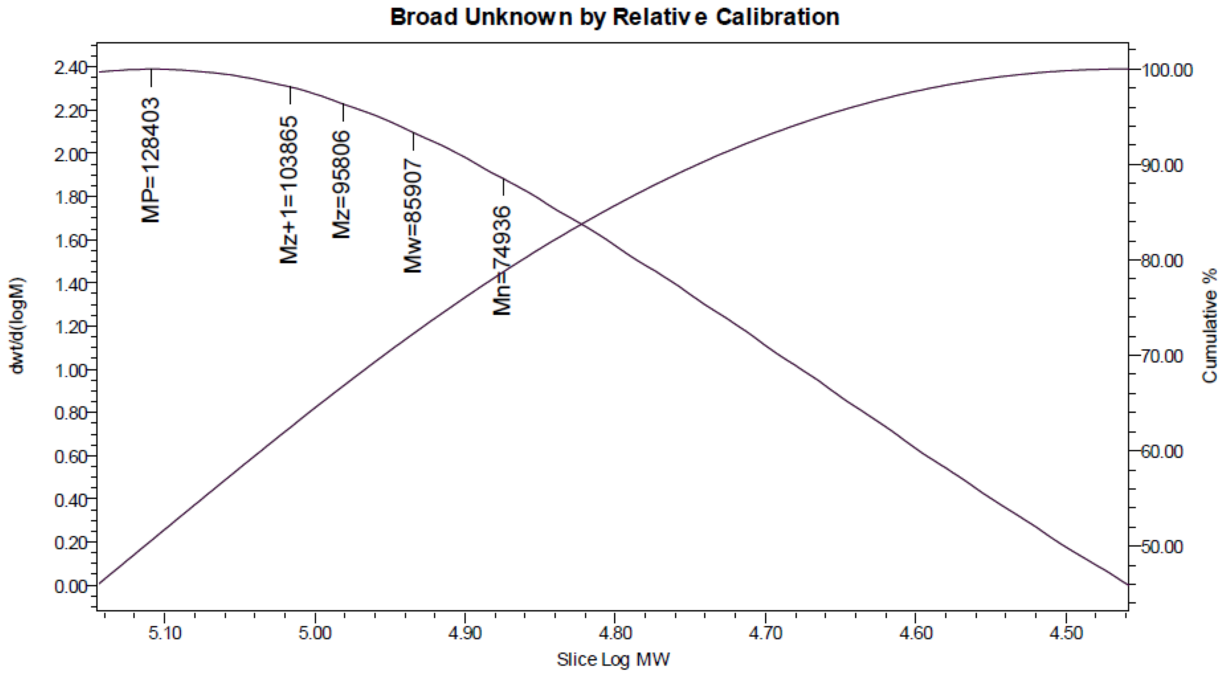

SF 4: TRP-3

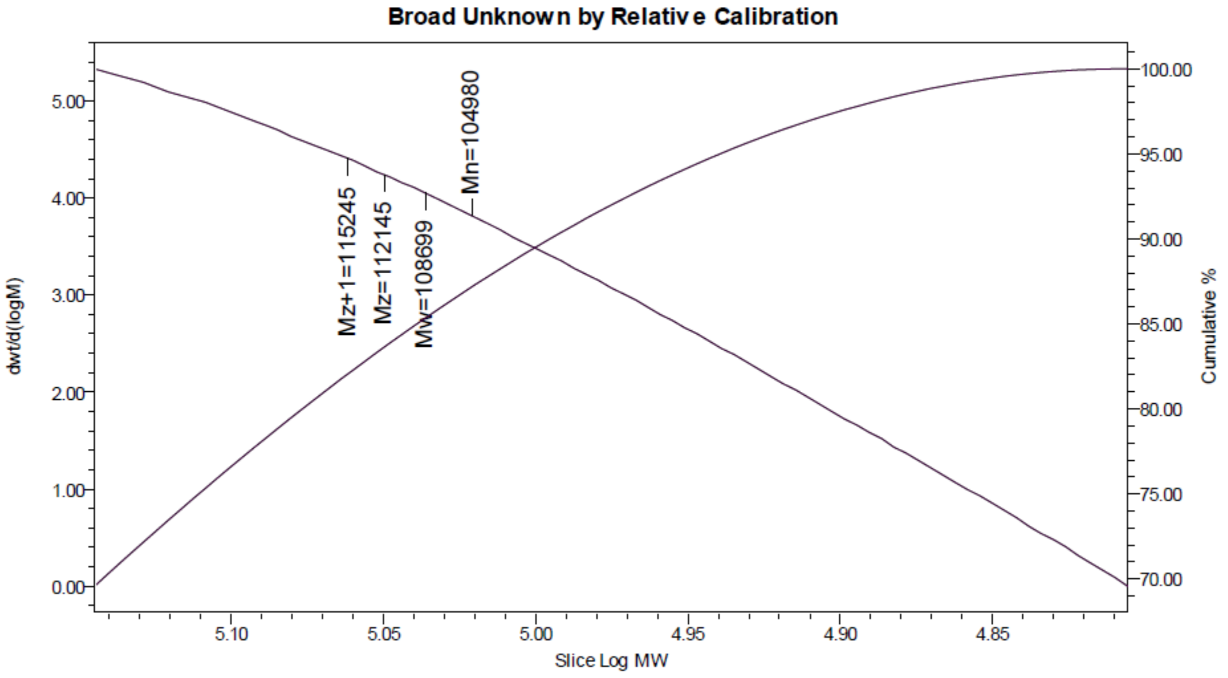

SF 5: TRP-4

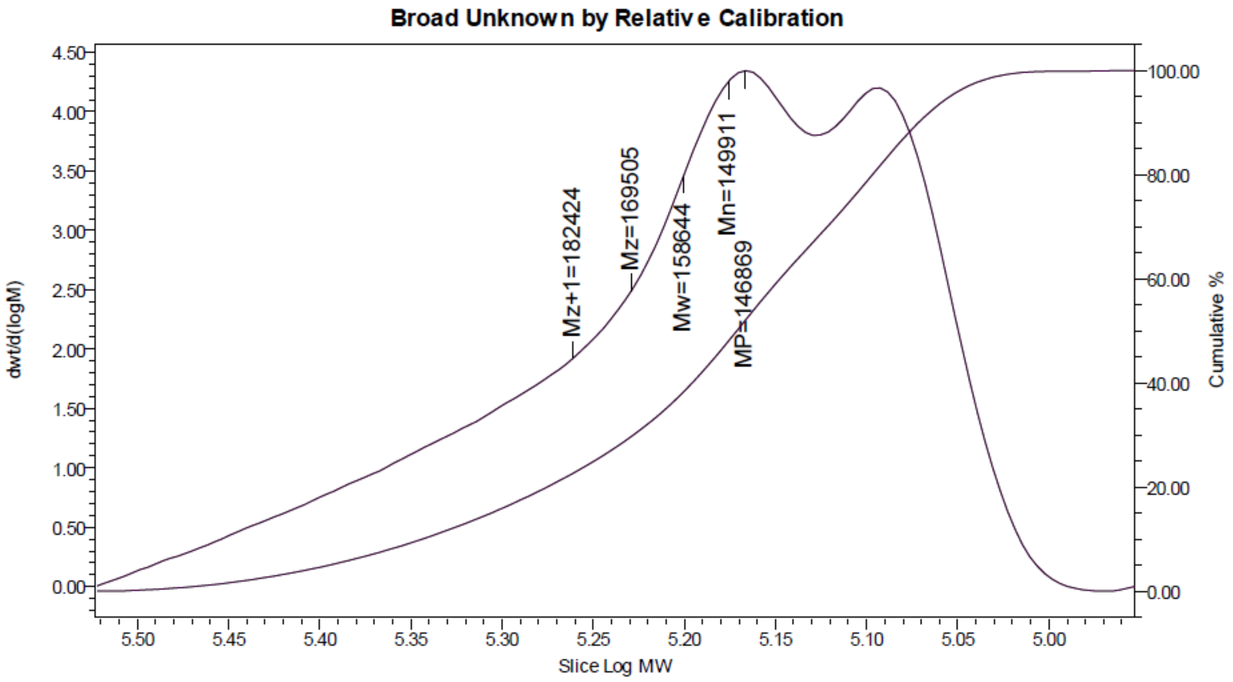

SF 6: TRP-5

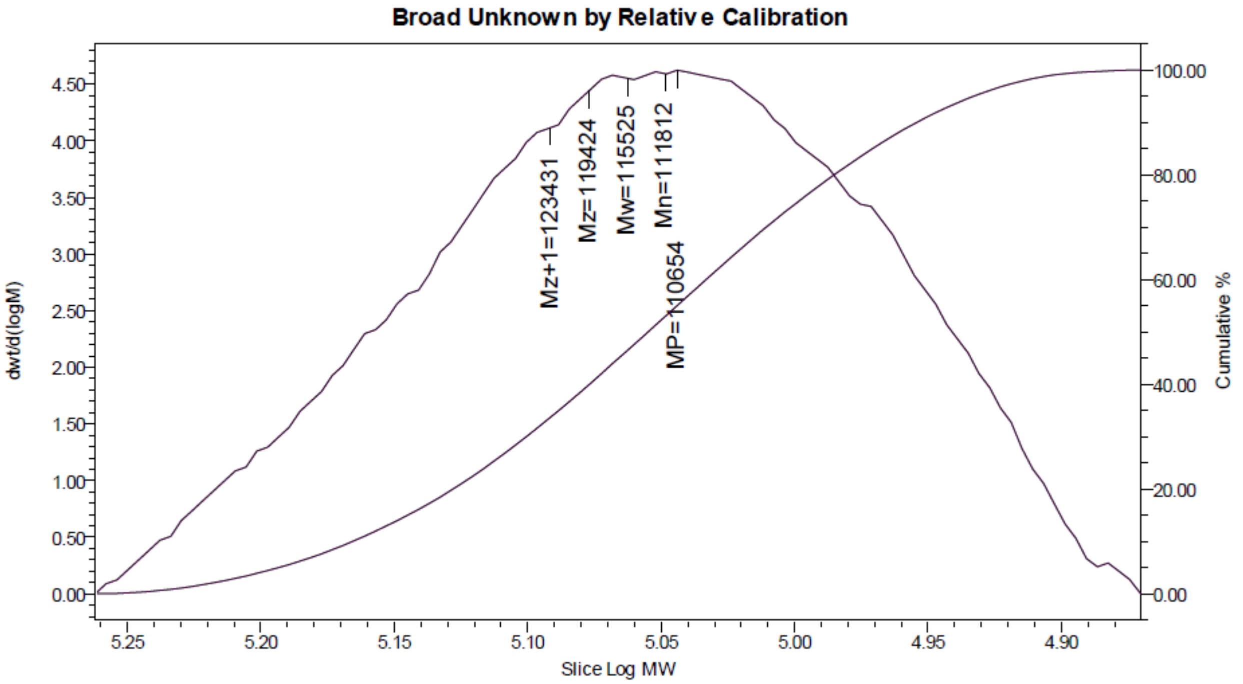

SF 7: β-CD-TRP-1

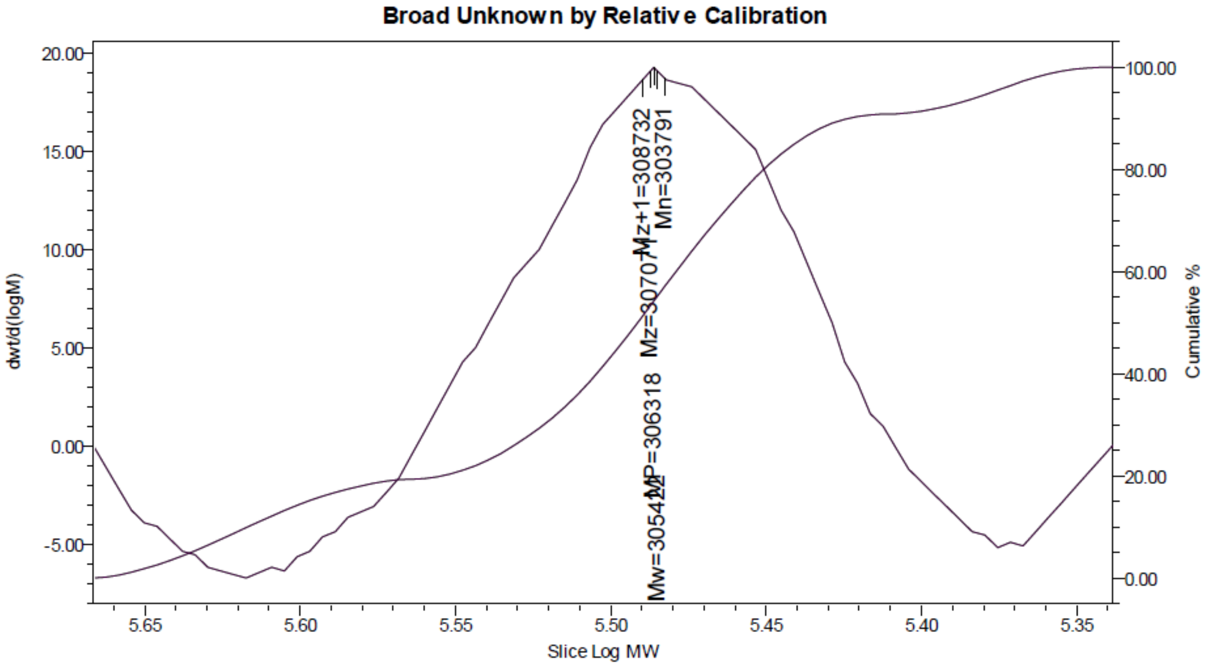

SF 8: β-CD-TRP-2

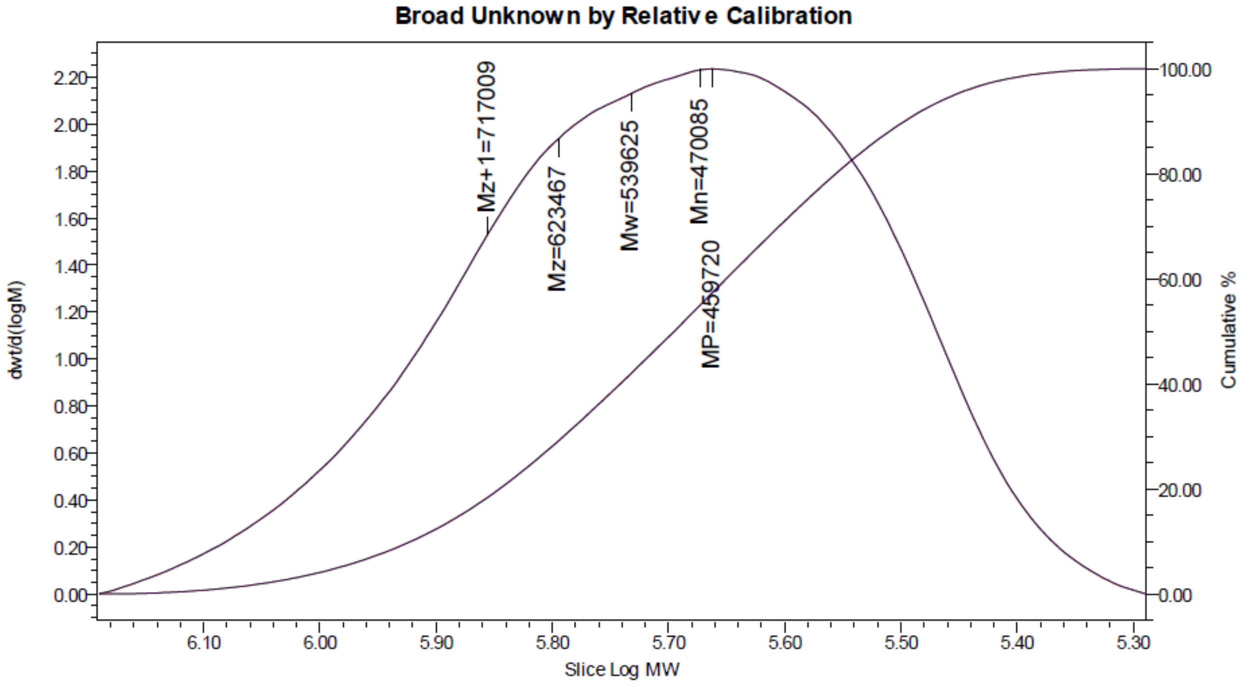

SF 9: β-CD-TRP-3

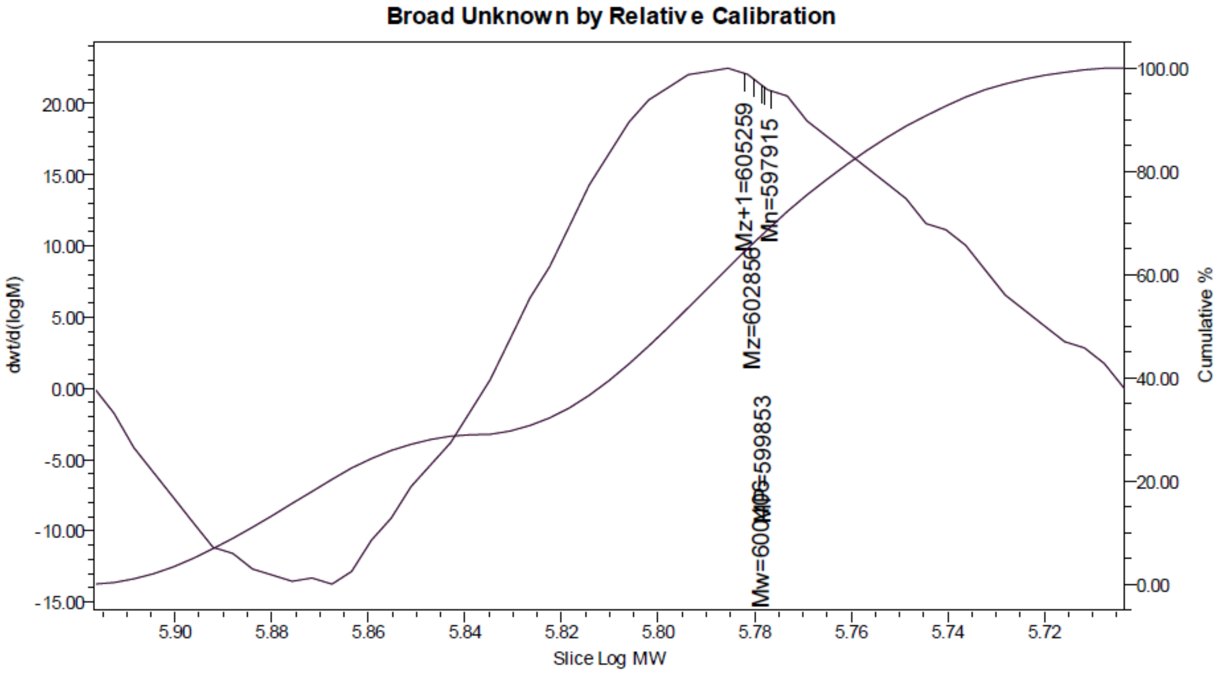

SF 10: β-CD-TRP-4

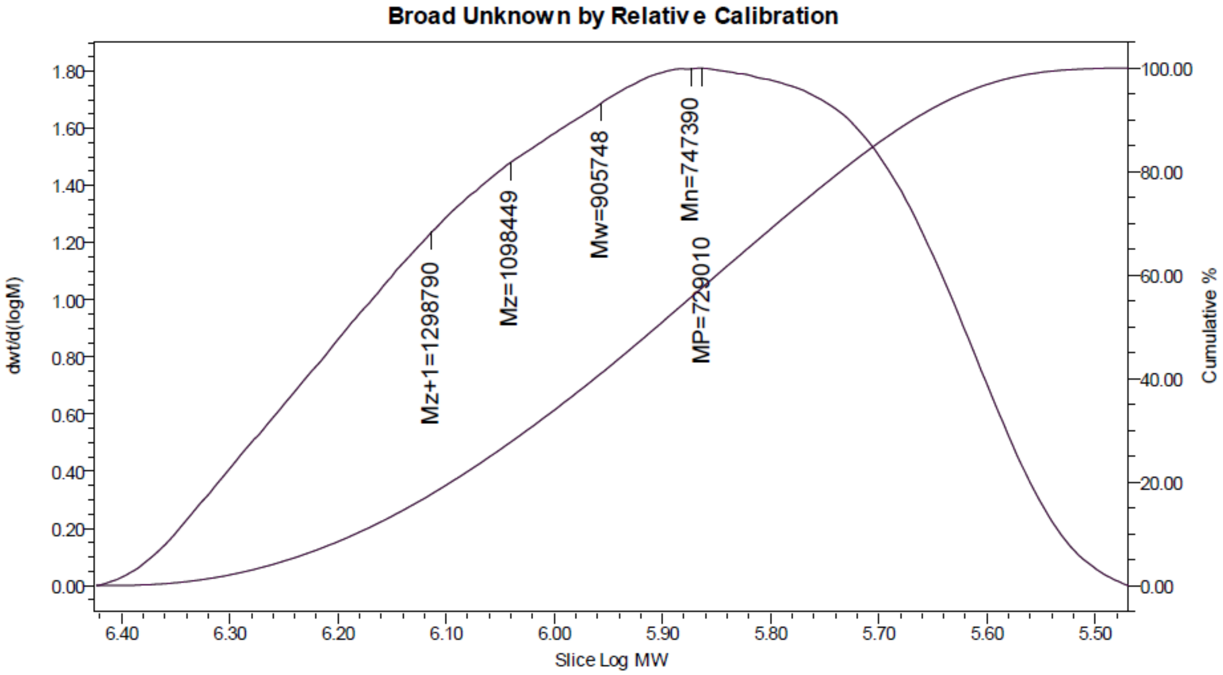

SF 11: β-CD-TRP-5

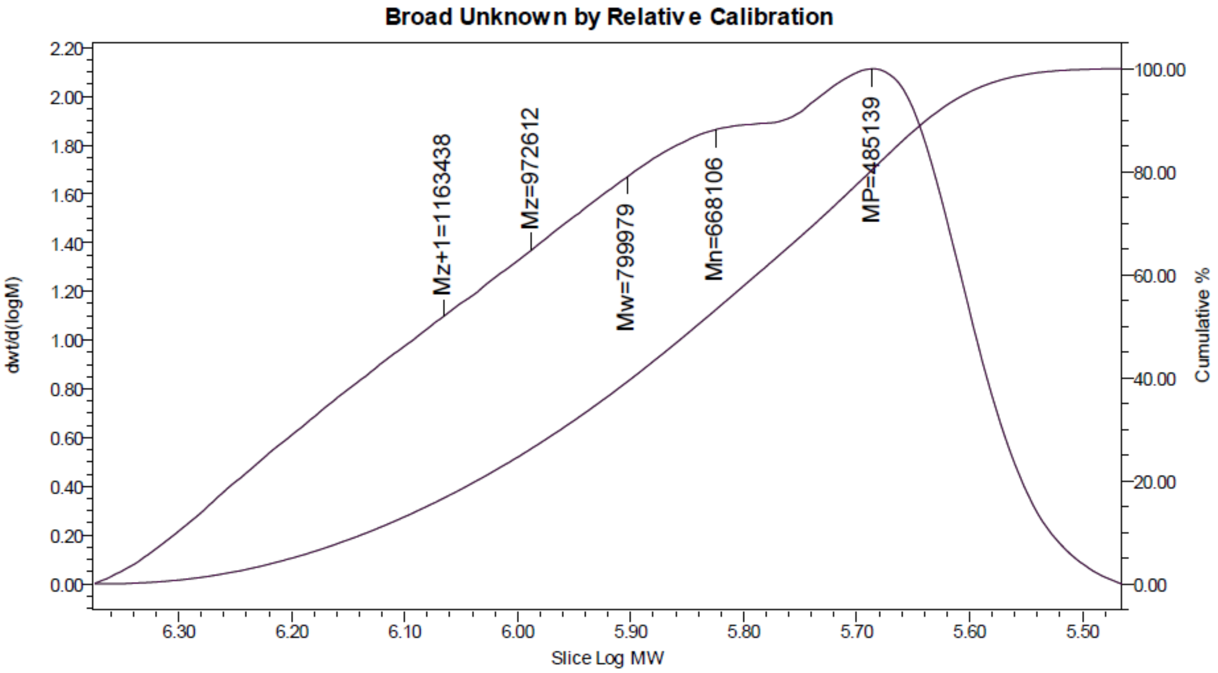

Supplement: Supplementary file 1 — Supporting Information [file GCH2-4-1900089-s001.pdf]
